# Efficacy and safety of pasireotide for Cushing's disease: A protocol for systematic review and meta-analysis

**DOI:** 10.1097/MD.0000000000023824

**Published:** 2020-12-18

**Authors:** Nairui Zhao, Xinxin Yang, Cuiliu Li, Jie Ma, Xiuping Yin

**Affiliations:** Second Department of Endocrinology, Cangzhou Central Hospital, Cangzhou, China.

**Keywords:** pasireotide, Cushing's disease, protocol, systematic review, meta-analysis

## Abstract

**Background:**

Cushing's disease (CD) is associated with increased risk of mortality, myocardial infarction, stroke, peptic ulcers, fractures and infections. The prevalence of CD is nearly 40 per million and higher in women than in men. When surgery has failed, is not feasible, or has been refused, pharmacotherapy can be considered a valuable option. Pasireotide is the first medical therapy officially approved for adult patients with CD. We will conduct a comprehensive systematic review and meta-analysis to systematically evaluate the efficacy and safety of pasireotide for CD.

**Methods:**

Five English databases (PubMed, Web of Science, Embase, Cochrane Library, and OVID) and 3 Chinese databases (China National Knowledge Infrastructure, China Science and Technology Journal Database, and Chinese Biomedical Literature Database) will be searched from their respective inception of databases to December 2020. Two reviewers will select articles, extract data and assess the risk of bias independently. Any disagreement will be resolved by discussion with the third reviewer. Review Manager 5.3 software will be used for data synthesis. The Cochrane risk of bias assessment tool will be used to evaluate the bias risk.

**Results:**

This systematic review and meta-analysis will conduct a comprehensive literature search and provide a systematic synthesis of current published data to explore the efficacy and safety of pasireotide for CD.

**Conclusions:**

This systematic review and meta-analysis will provide clinical evidence for the efficacy and safety of pasireotide for CD, and inform our understanding of the value of pasireotide in improving CD clinical signs and symptoms. The conclusions drawn from this study may be beneficial to patients, clinicians, and health-related policy makers.

**Study registration number:**

INPLASY2020110070.

## 1 Introduction

Cushing's disease (CD) is caused by pituitary adenomas that secretes adrenocorticotropic hormone (ACTH) and represents the most common cause of endogenous Cushing's syndrome.^[1,2]^ The prevalence of CD is nearly 40 per million and higher in women than in men.^[3,4]^ Clinical manifestations mainly includes weight gain, moon face, hypertension, lower limb edema, impaired glucose tolerance or type 2 diabetes, osteopenia, hyperpigmentation of the skin and mucous membranes, depression and so on.^[5–8]^ CD is associated with increased risk of mortality, myocardial infarction, stroke, peptic ulcers, fractures and infections.^[9,10]^ Pituitary surgery is the first-line treatment for most CD patients.^[11]^ When surgery has failed, is not feasible, or has been refused, pharmacotherapy can be considered a valuable option.^[12]^ Many patients require long-term medication to induce disease remission. Ketoconazole, metyrapone and mitotane are the most commonly used medical therapies, and target the adrenal glands.^[13–15]^ These drugs do not treat the underlying cause of the disease and restore normal function of the hypothalamo-pituitary-adrenal axis.^[16,17]^

Pasireotide, a multireceptor-targeted somatostatin analogue, is the first medical therapy officially approved for adult patients with CD.^[18]^ It has a high affinity for somatostatin receptor subtype 5 that causes a decrease in cyclic adenosine monophosphate and increase in potassium efflux, resulting in decreased cyclic AMP formation and ACTH secretion.^[19–21]^ In recent years, pasireotide has been shown to improve clinical signs and symptoms of CD, provide sustained reductions in mean urinary free cortisol (mUFC), reduce cardiometabolic risk factors, and increase CD patients life quality with no new safety signals emerging.^[22–25]^

While up to now, no systematic review or meta-analysis has been performed on the efficacy and safety of pasireotide for CD. Therefore, we will conduct a comprehensive systematic review and meta-analysis to systematically evaluate the effects of pasireotide for CD and provide more scientific evidence for clinical strategy.

## 2 Methods

### 2.1 Study registration

This protocol for systematic review and meta-analysis has been on INPLASY (INPLASY2020110070). It has been drafted under the guidance of the Preferred Reporting Items for Systematic Reviews and Meta-Analyses (PRISMA) statement.^[26]^

### 2.2 Eligibility criteria for study selection

#### 2.2.1 Types of studies

All available randomized controlled trials (RCTs) of pasireotide for CD will be included, while case reports, animal experiments and reviews will be excluded.

#### 2.2.2 Types of participants

Participants who are diagnosed with CD regardless of nationality, age, gender, and race will be included.

#### 2.2.3 Types of interventions

In treatment group, patients were given pasireotide with no limitations of administration routes, dosage or duration of intervention.

In control group, patients were given conventional treatments, placebo therapy, or no treatment.

#### 2.2.4 Types of outcomes

The primary outcome contains mUFC and the percentage of cortisol normalization. Mean fasting glucose, glycated haemoglobin and drug-related adverse events will be designated as the secondary outcome.

### 2.3 Search strategy

Five English databases (PubMed, Web of Science, Embase, Cochrane Library, and OVID) and 3 Chinese databases (China National Knowledge Infrastructure, China Science and Technology Journal Database, and Chinese Biomedical Literature Database) will be searched from their respective inception of databases to December 2020. Additional trials will be searched by reviewing the reference lists of the retrieved articles, conference proceedings, and gray literature. The detailed search strategy for PubMed is shown in Table 1. The similar search strategies will be used for other electronic databases.

**Table 1:** Search strategy for PubMed.

Number	Search terms
1	Cushing's disease
2	Cushing disease
3	Cushing's syndrome
4	hypercortisolism
5	Cortisol
6	hydrocortisone
7	Or 1–6
8	Pasireotide
9	somatostatin analogue
10	somatostatin receptor subtype 5
11	SSRS5
12	Or 8–11
13	randomized controlled trial
14	clinical trial
15	RCT
16	Or 13–15
17	7 and 12 and 16

### 2.4 Selection of studies

The searched literature will be integrated into Endnote X9. After excluding duplicates by Endnote X9, 2 reviewers will independently check the titles and abstracts for each retrieved record according to the eligibility criteria. The remaining records will be read by full-texts, and then final included studies will be determined. Any disagreement regarding the study selection between 2 reviewers will be resolved by the third reviewer. A PRISMA flow diagram will be designed to describe the details of selection process.

### 2.5 Data extraction and management

Using a standardized data collection form, 2 reviewers will abstract the following information from each study: authors name, publication date, journal, study design, summary of baseline characteristics of participants, number of participants in each arm at study onset and completion, experimental intervention, control intervention, and outcomes. Disagreement will be solved by the third reviewer. If some required items are not shown in the published literature, we will contact the corresponding author of original study for detailed information by e-mail.

### 2.6 Assessment of risk of bias

The Cochrane risk of bias assessment tool will be used by 2 reviewers independently to evaluate the bias risk of the subsequent areas of all included studies: random sequence generation, allocation concealment, blinding of participants and personnel, blinding of outcome assessment, incomplete outcome data, selective reporting and other bias. Disagreement will be solved by the third reviewer. Bias graph and graphic summary of risk of bias will be provided in the completed review.

### 2.7 Data synthesis and analysis

#### 2.7.1 Data synthesis

Review Manager 5.3 software will be used for data synthesis. Standardized mean difference or mean difference with 95% confidence interval will be used for continuous variables, and risk ratio with 95% confidence interval will be used for dichotomous variables. We will use *I*^2^ test to identify heterogeneity. The *I*^2^ value >50% means significant heterogeneity, and the random effects model will be utilized. The *I*^2^ value ≤50% means acceptable heterogeneity, and the fixed effects model will be utilized.

#### 2.7.2 Subgroup analysis

Subgroup analysis will be carried out based on different participant characteristics, administration routes, dosage and duration of intervention, and outcome assessments to explore any possible sources of significant heterogeneity among included trials.

#### 2.7.3 Sensitivity analysis

Sensitivity analysis will be carried out to test the robustness and stability of data analysis by repeated meta-analysis after eliminating low quality studies.

#### 2.7.4 Reporting bias

If over 10 trials are included, we will carry out funnel plot and Egger regression analysis to examine the publication bias.^[27,28]^

### 2.8 Ethics and dissemination

Ethical approval is not necessary because this study is based on literature analysis. The results of this study will be published in a peer-reviewed journal.

## 3 Discussion

To our best knowledge, this is the first systematic review and meta-analysis to investigate the efficacy and safety of pasireotide for CD. Five English databases and 3 Chinese databases will be searched to avoid missing any potential eligible studies. We will apply rigorous methodology to examine studies reporting pasireotide for CD. This systematic review and meta-analysis will provide clinical evidence for the efficacy and safety of pasireotide for CD, and inform our understanding of the value of pasireotide in improving CD clinical signs and symptoms. We believe that the conclusions drawn from this study may be beneficial to patients, clinicians, and health-related policy makers.

## Author contributions

**Conceptualization:** Nairui Zhao, Xinxin Yang.

**Data curation:** Nairui Zhao, Xinxin Yang, Cuiliu Li.

**Formal analysis:** Nairui Zhao, Jie Ma, Xiuping Yin.

**Investigation:** Xinxin Yang, Cuiliu Li, Jie Ma.

**Methodology:** Xinxin Yang, Xiuping Yin.

**Project administration:** Nairui Zhao.

**Resources:** Nairui Zhao, Xinxin Yang, Cuiliu Li.

**Software:** Cuiliu Li, Jie Ma, Xiuping Yin.

**Supervision:** Nairui Zhao.

**Validation:** Nairui Zhao, Xinxin Yang.

**Visualization:** Cuiliu Li, Jie Ma, Xiuping Yin.

**Writing – original draft:** Nairui Zhao, Xinxin Yang, Cuiliu Li.

**Writing – review & editing:** Nairui Zhao, Xinxin Yang, Cuiliu Li, Jie Ma, Xiuping Yin.
